# Supplementary material for: Estimating Regional Methane Emission Factors from Energy and Agricultural Sector Sources Using a Portable Measurement System: Case Study of the Denver–Julesburg Basin
Source: Sensors (Basel). 2022 Sep 29;22(19):7410. doi: 10.3390/s22197410 (PMC9572259; doi:10.3390/s22197410)
Supplement: Supplementary file 1 [file sensors-22-07410-s001.zip › sensors-1916932-supplementary.pdf]

Supplementary Information

Estimating regional methane emission factors from energy and agricultural sector sources using a portable measurement system: Case study of the Denver-Julesburg Basin

Supplementary Information 1: Driving survey sampling point and area sources

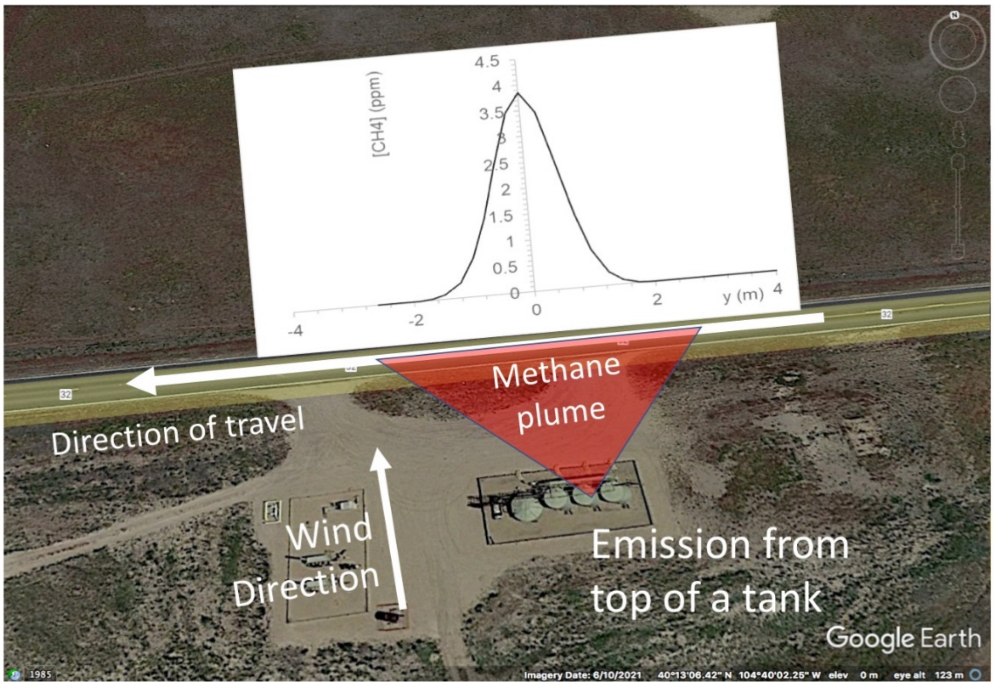

Figure S1 Driving survey sampling point emission

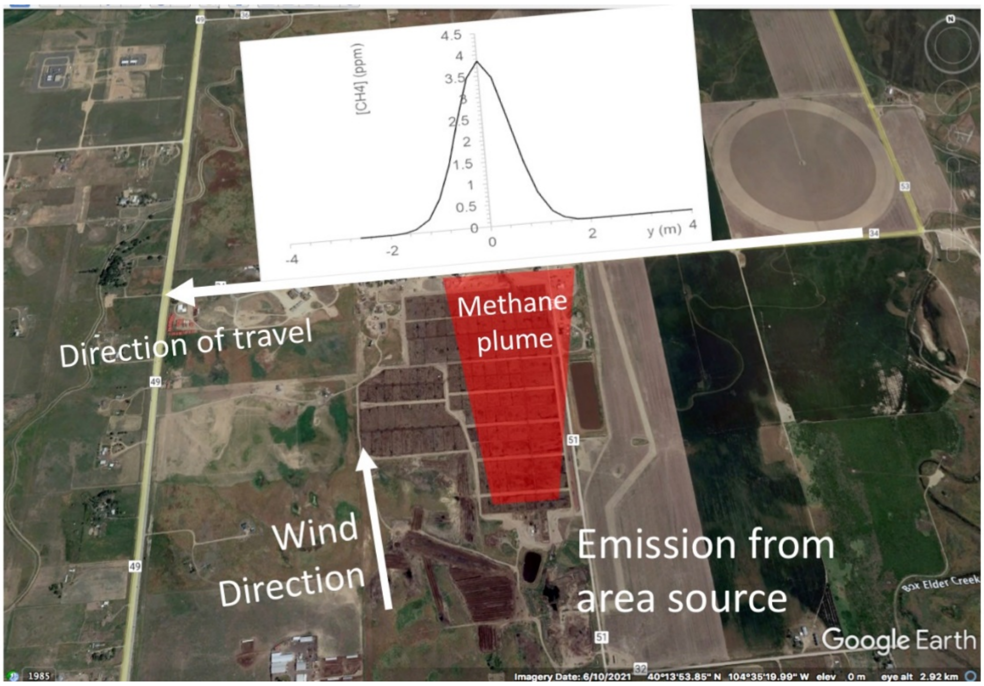

Figure S2 Driving survey sampling area emission

Supplementary Information Section 2—Pasquill Gifford Stability Classes (PGSC)

Estimating the Pasquill-Gifford Stability Class from wind speed and irradiance (Table S1). Pasquill and Smith (1983) define strong isolation as sunny midday in midsummer in England and slight insolation to similar conditions in midwinter. Strong irradiance as > 1 kW m^{-2}, moderate irradiance 0.5 kW m^{-2} to 1 kW m-2 and light irradiance as > 0.5 kW m^{-2}.

Table S1 Pasquill Gifford Stability Class lookup table

Stability Class			
Wind Speed (m s^{-1})	Strong	Moderate	Slight
2	A	A	B
3	B	B	C
4	B	C	C
5	C	C	D
6	C	D	D

Supplementary Information Section 3—Example emission calculations for point and area sources

Point sources

Table S2 Data input to Equation 1 to calculate methane emission from a point source

x (m)	y (m)	z (m)	u (m s^{-1})	PGSC	σ_y (m)	σ_z (m)	h_s (m)	h (m)	X (µg m^{-3})	Q (kg hr^{-1})
65	0	1.5	1.9	A	18.94	1.56	0.5	5000	1.11	0.89

Area sources

Table S3 Data input to WindTrax to calculate methane emission from an area source

x (m)	y (m)	z (m)	u (m s^{-1})	WD (°)	PGSC	Width of source (m)	Length of source (m)	X (mg m^{-3})	Q (mg m^{-2} s^{-1})	Q (kg hr^{-1})
50	0	1.5	4.5	5.4	B	300	300	0.60	0.140	45.5
